# A Comparison of Numerical and Actual Measurements of Large-Scale Rib-Structured Pallet Flatness Using Recycled Polypropylene in Injection Molding

**DOI:** 10.3390/polym14081631

**Published:** 2022-04-18

**Authors:** Yi-Ling Liao, Hsi-Hsun Tsai

**Affiliations:** Department of Mechanical Engineering, Ming Chi University of Technology, New Taipei City 24301, Taiwan; yiling@mail.mcut.edu.tw

**Keywords:** plastic pallet, injection molding, flatness, sequential valve gate system, molding flow analysis

## Abstract

Many challenges are associated with the injection molding process for forming a rib-structured pallet (1100 mm × 1100 mm × 140 mm, length × width × height) because greater flowing resistance through the rib channels within the pallet can induce insufficient filling. Essentially, multi-gate filling involves a sequential valve gate system, which helps to spread the filling front with fewer weld lines. Based on the presetting of the sequential scheme of the valve gates, actual measurements of pallet flatness using the ATOS scan system were compared to numerical warpage measurements of a pallet derived by Moldex3D 2020. In this study, we propose a sequential scheme by actuating the valve gates to open once the flow front spreads towards them; then, actual warpage measurements of a pallet are compared with numerical measurements. The results show that the warpage of the top surface of the pallet is 5.144 mm in actual measurements and 5.729 mm in simulation. The results all indicated small warpage with respect to the pallet size. The simulation and actual measurements of flatness are in excellent agreement; the difference in top flatness between the simulated and actual pallet is 0.59 mm, while the bottom flatness difference is 0.035 mm. By adjusting the cooling water temperature, increasing the mold temperature, and decreasing the material temperature, overall flatness and warpage displacement can be reduced.

## 1. Introduction

Recently, given thin-wall features of plastic components, the injection molding process has become one of the major manufacturing technologies used in the modern plastics industry. During thin-wall injection molding, melted material under high pressure flows into a mold cavity using screws and barrels to overcome melt flow resistance within the thin-wall features of the mold cavity. During the filling stage, the flow front of the melt polymer spreads and fills the cavity, while the freezing of the melt flow front must be avoided. However, the mold temperature is relatively low as compared with the melt polymer, and therefore, heat from the flow front of the melt polymer is concurrently transferred to the mold plates and the cooling channels. Therefore, the length of the melt flow is limited at each filling gate due to the variety of connection features in the thin wall and ribs within the mold cavity. Increasing the mold temperature or filling pressure can enhance the length of the melt flow. The temperature in the cooling channels can elevate the mold temperature and induce a longer delay time, and thus, a longer cycle time in the injection molding process. The cycle time is the first concern in the injection molding process, which is negatively proportional to the production cost. Hence, an external induction heating system can efficiently increase the length of the melt flow, thanks to an electro-magnetic heating process, which can heat the plate to 290 °C within 5 s [1]. An external heating approach using gas from 200 to 400 °C can also be utilized to heat the mold temperature, increasing the length of the melt flow [2].

Nevertheless, by using an external heating approach to increase mold temperature, there are still limitations associated with the length of the melt flow from a filling gate. For large-size molding components, multiple filling gates are essential to fulfill the molding requirements. However, when melted plastic materials from multiple filling gates mix with each other, they form a weld line, the strength of which is less than the general plastic molding material, since the temperature of the mixing at the weld line is decreased [3]. A large plastic pallet with several thin walls and rib features would be molded using multiple injection gates for filling the melted polymers, with a high injection rate and pressure. The weld lines within the plastic pallet may have the potential for failure under loading. To eliminate or decrease the number of weld lines caused by multiple filling gates, a sequential valve gate system approach associated with a hot runner system can be utilized in plastic pallet injection molding. By dividing the filling gates into several groups, the gates of the second group are actuated to fill once the flow fronts of the first group spread to meet them. A sequential valve gate system can eliminate weld lines and decrease the clamping and packing forces in the injection molding process [4]. Fewer weld lines within large plastic pallets can also accommodate higher loads for the transport of stacked products by a forklift.

Initialized by the finite volume method to discretize the Navier–Stokes equation, Moldex3D would solve the transient flow field in complex three-dimensional geometry under a pressure-based decoupled procedure. An iterative decoupled procedure for coupling velocity and pressure was also utilized to derive flow field, and the three linearized momentum equations were therefore solved. The pressure correction equation was iterated. The modified Cross model with Arrhenius temperature dependence is assumed to be the viscosity of the melting polymer. The velocity and temperature are specified at the mold sprue during the filling stage [5]. The injection molding process involves multiple physical parameters of the output molding components in relation to the injection parameters. Moldex3D is one of the commercialized software programs for analyzing multiphysics problems in injection molding. Successful investigations in injection molding by Moldex3D have been reported [6,7,8,9,10,11,12,13,14]. The numerical and actual measured warpages of a buy/sell type [15] of a recycled PP pallet via a sequential valve gate system were compared by simulations with Moldex3D and the actual profiles using the scan box 5120 system (GOM, Swiss, www.gom.com, accessed on 9 February 2022), which is a non-contact three-dimensional measuring system that operates via high-speed sensors used for scanning all the parts. The study found that the temperature of the cooling channel within the cavity plate should be higher than the temperature within the core plate, which could decrease the height directional warpage to about 7 mm. Based on the melt flow fronts from value gates by a Moldex3D simulation, the time sequence of the valve gates could also help to decrease the number of weld lines [13]. Using Moldex3D, the authors numerically compared the warpages of a pallet with a sequential valve gate system and a concurrent valve gate opening system and found that the sequential valve gate system resulted in greater warpage of the pallet. They also found that the longitudinal warpage of the pallet was negatively proportional to the cooling time. The top and bottom flatness both became smaller before 28 s of cooling time, and then were the worst after 28 s [14]. In their results, a cooling time of 28 s gave a minimal flatness of the pallet, which differed from the empirical prediction.

The optimization of the injection molding parameters for achieving a specific index of quality of the molded parts is essential in polymer processing technologies [15]. To effectively confirm the numerical and experimental results of molding a rib-structured buy/sell type pallet, in this study, we aim to experimentally investigate the actual warpages of a pallet with the dimensions of 1100 mm × 1100 mm × 140 mm using the ATOS scan box 5120 system and then to compare the actual measurements with the numerical warpages of a pallet derived by Moldex3D 2020. By using the injection parameters of the recycled PP pallet, the pre-setting sequence applied to the valve gates provided by the cooperating company, and the specifications of the injection machine, as well as the recycling database, a fundamental investigation was conducted to understand the effects on pallet injection molding. Then, we propose a newly derived sequential valve gate scheme to improve the flatness of the pallet, which can help to evaluate the accuracy of numerical predictions.

## 2. Experimental Setup and Software

**Figure 1:**
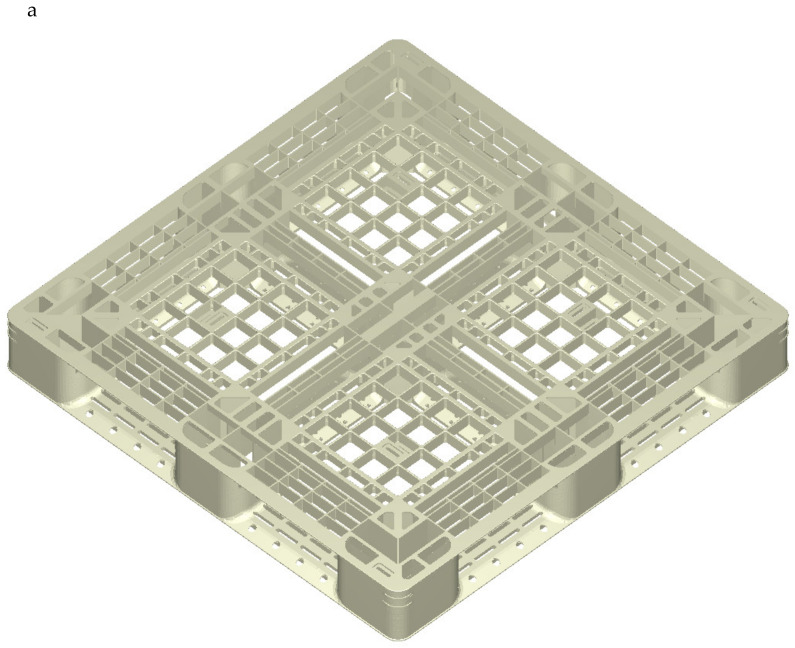

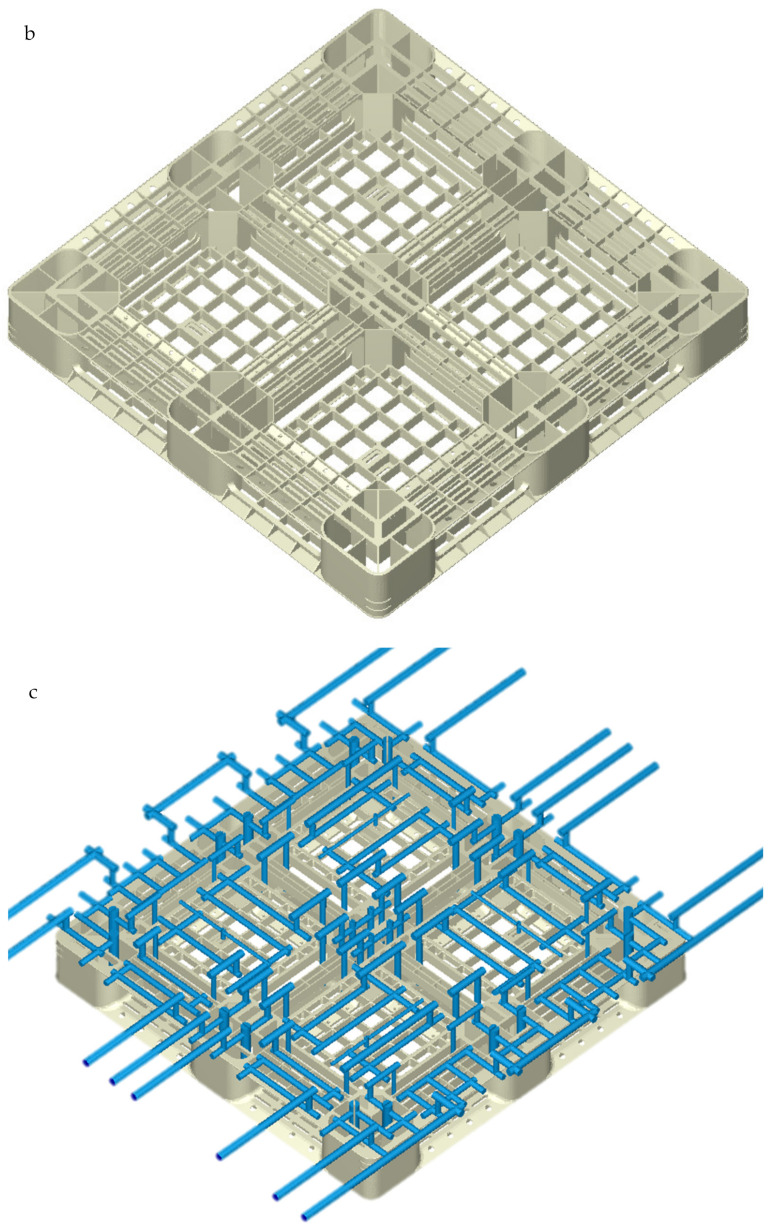

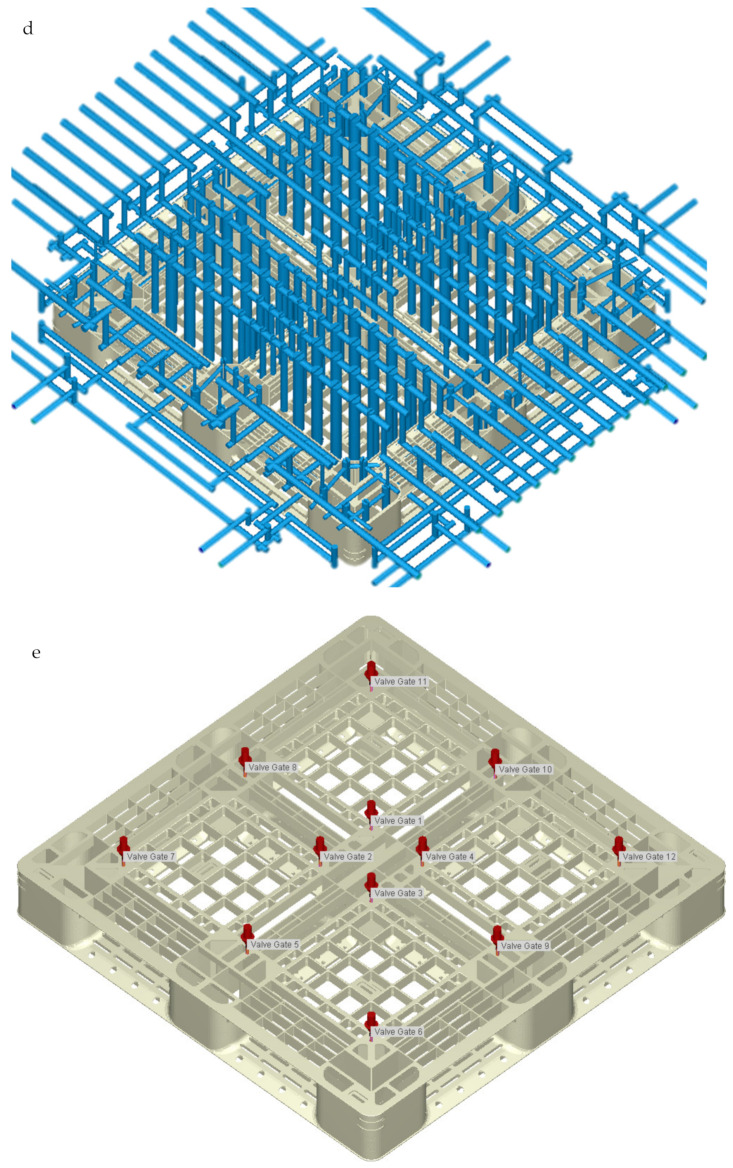
Injection-molded pallet (1100 mm × 1100 mm × 140 mm): (**a**) isometric view of the pallet (top side); (**b**) isometric view of the pallet (bottom side); (**c**) isometric view of the baffle cooling flow system on the top side of the pallet; (**d**) isometric view of the baffle cooling flow system on the bottom side of the pallet; (**e**) 12 filling gates in the pallet.

Figure 1a,b shows the top view and bottom view of a buy/sell type plastic pallet, respectively. The reinforced structure is filled with ribs and thin walls; the ribs are 2.0 mm in thickness and are changed according to the draft angle used for the injection molding. The dimensions of the pallet are 1100 mm in length and width and 140 mm in height. Figure 1c shows an isometric view of the complex features of the cooling flow system on the top of the pallet, including dedicated straight and baffle cooling tubes required during the cooling stage in injection molding. Figure 1d shows an isometric view of the cooling flow system on the bottom of the pallet, showing that the cooling channel system contains more baffle tubes within the core plate. An empirical rule for the positioning of the filling gates of the mold is to ensure the ratio of melt flow length to rib thickness is below 150. The running length of the melting polymer during the filling stage is sufficient to maintain the melting state once the ratio is lower than 150. This pallet requires twelve filling gates. Each gate has a 5.0 mm diameter and a 20 mm height, as shown in Figure 1e.

This pallet has the sizes of 1100 mm in length and width and 140 mm in height. The volume of the plastic pallet is 13,160,000 mm^3^, and the mesh of the plastic pallet without cooling geometry contains 2,775,565 elements of the boundary layer mesh. The material properties of the recycled PP include: a density of recycled PP of 1026 kg/m^3^; a true ultimate strength of 45.2 MPa; thermal conductivity of 0.226 W/(m°C); and melt and freeze temperatures of 250 and 117 °C, respectively [14]. In Table 1, injection molding parameters of recycled polypropylene are obtained from the previous study [14]. A Supermaster 3600E1 molding machine (https://chenhsong.com/, Taoyuan Taiwan, assessed on 16 February 2022) was implemented in this study, which has a maximum injection pressure of 102 MPa for the melt polymer, a screw diameter of 225 mm, a maximum injection volume of 49,278 cm^3^, a maximum injection rate of 2475 g/sec. for the melt polymer, a maximum clamp force of 3600 tons for the mold base, and a screw stroke within the barrel of 1240 mm.

**Figure 2:**
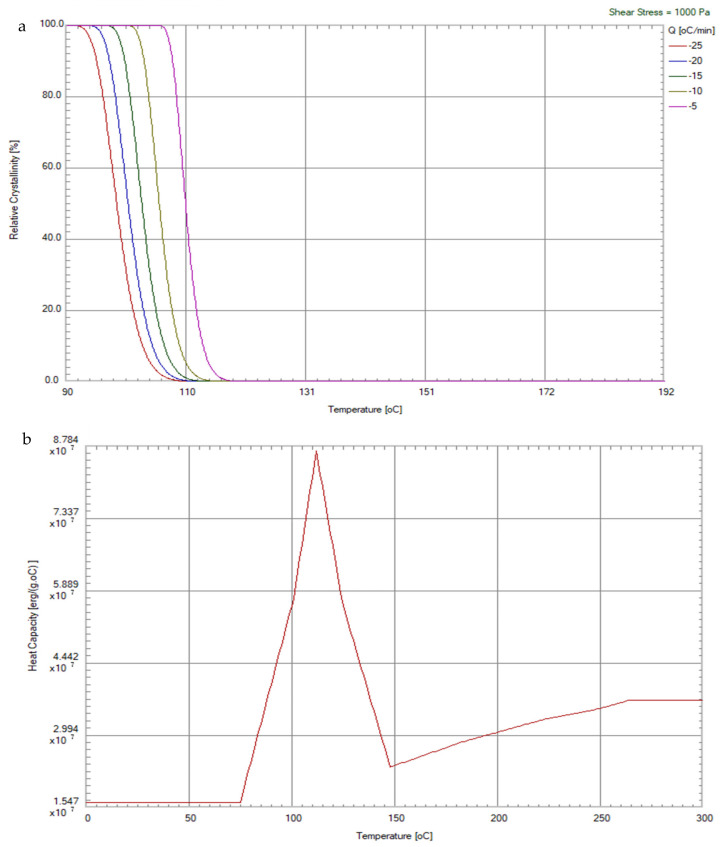

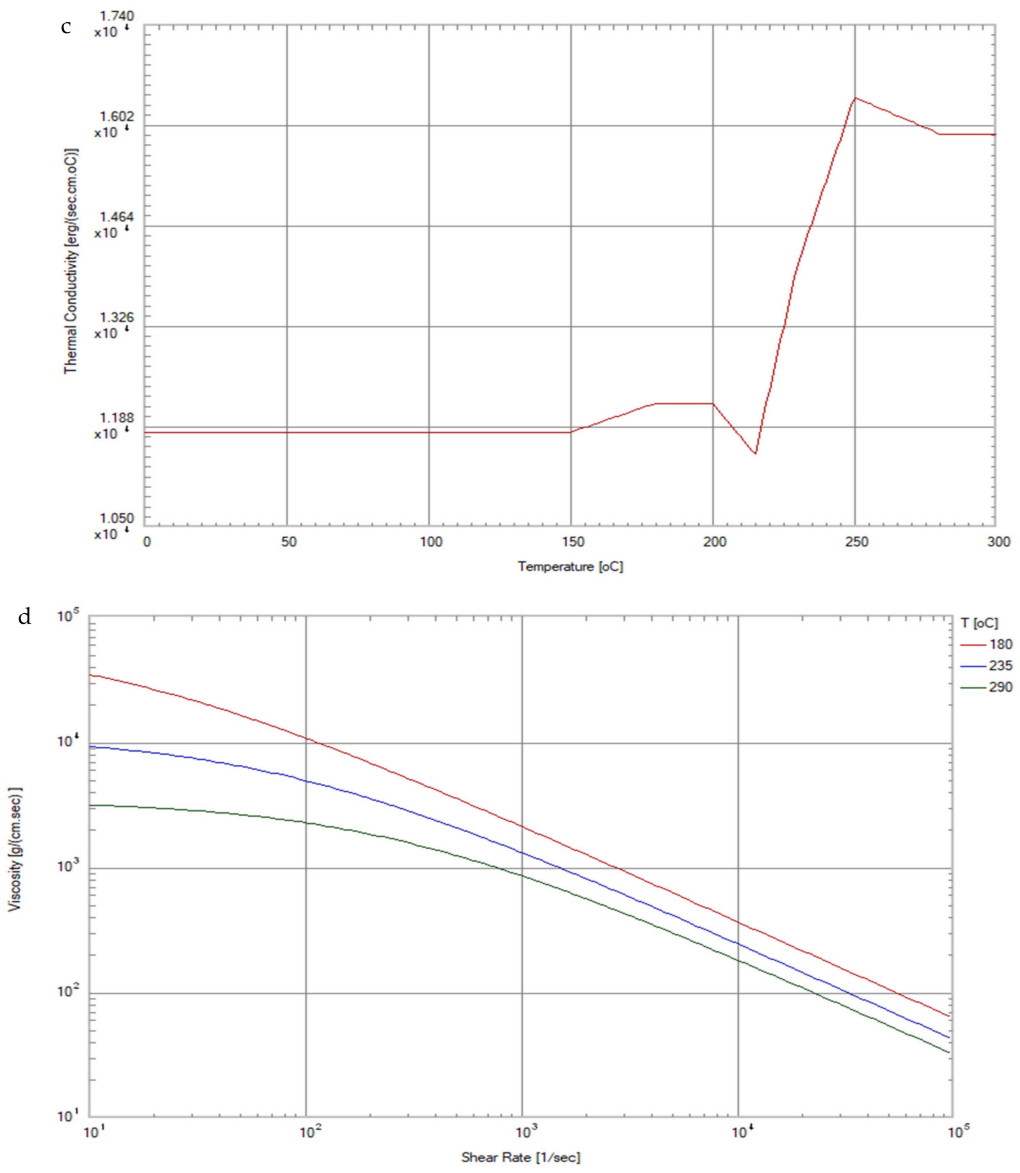

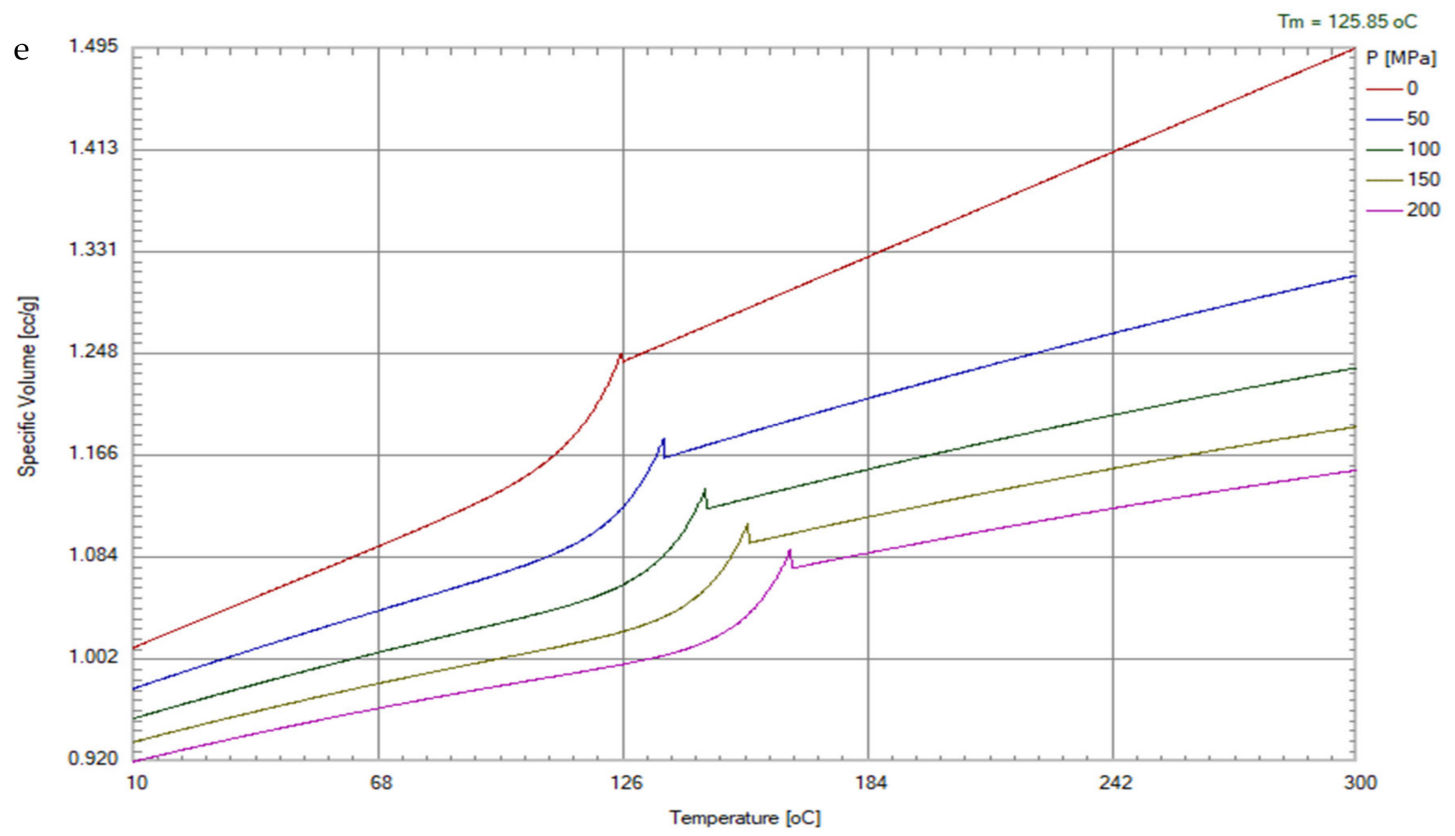
Properties of recycled PP: (**a**) relative crystallinity with respect to shear rate and cooling rate; (**b**) constant pressure heat capacity with respect to temperature; (**c**) thermal conductivity with respect to temperature; (**d**) viscosity with respect to temperature; (**e**) pressure–volume–temperature relationships.

This material was provided and material properties were measured by the Nan Ya Plastic Corp., Taipei, Taiwan. As depicted in Figure 2a, a thermal differential scanning calorimeter (DSC) from Perkin Elmer DSC-8500 was used to measure the curing kinetics of recycled PP. The constant pressure specific heat and thermal conductivity of the recycled PP were also measured by DSC in Figure 2b,c. A parallel-plate rheometer (Anton Paar MCR502) was used to measure the viscosity of the recycled PP. The viscosity of a melt polymer is inversely proportional to the temperature and shear rate, as shown in Figure 2d. Pressure–volume–temperature (PVT) properties of the amorphous and semi-crystalline polymers are important for polymer processing engineering. A GOTECH PVT-6000 L was therefore implemented to measure the PVT of recycled PP, as shown in Figure 2e.

The preset sequence of the sequential valve gate scheme from the corporation that is funding the research is depicted in Table 2. According to Table 2, phase 1 involves opening gates 1–4 and 10 for 6 s before closing. Phase 3 occurs after 6 s, when gates 5 and 9 are opened to continue the filling process for 3 s before closing. Phase 4 occurs at 8 s, when gate 8 resumes the filling process. Phase 5 occurs at 9 s, when gates 5, 9, and 10 are closed. Then, gates 5, 9, and 10 are opened at 9.7 s, 9.8 s, and 9.9 s, respectively. The final phase occurs at 12 s, when gates 6, 7, 11, and 12 are opened to sustain the filling process until completion.

Using a Supermaster 3600E1 molding machine for experiments, the gates’ opening and closing times are controlled sequentially via the pneumatic system, as listed in Table 2. Under the sequential valve gate scheme, the hot runners are opened at different times. The fill flow front of the molten material is closely dependent on the viscosity during the filling stage. The flow front is controlled by the filling rate and pressure gradient on the gate. The rPP pallet is 1 m in length and width and 0.13 m in height. This scale of pallet is too large to be measured by an ATOS scan box 5120 system (GOM, Swiss, www.gom.com, 9 Febuary 2022). The actual pallet was measured by RATC in Taiwan (https://www.ratc.com.tw/, accessed on 9 February 2022) with the ATOS scan box 5120. The measured flatness of each surface on the pallet is compared with the numerical results derived by Moldex3D.

## 3. Theoretical Models of Multiphysics in Injection Molding

For the simulation of molten plastic within the mold cavity associated with the runner and gate in the injection molding process, the principles of conservation of mass during the filling stage, conservation of momentum during the filling stage, and conservation of energy of the flowing molten polymer during the filling, packing, and cooling stages were implemented for governing the equations. The momentum equation expresses the law of conservation of momentum for moving melted polymer. The rate of change in total momentum of any micro cubic element in the flow field is equal to the resultant force of all external forces acting on the micro element. For a Newtonian fluid, the partial differential equations in the vector expression of the fluid momentum equation is as follows [16]:

(1) $$\frac{\partial (\rho \vec{V)}}{\partial t}+\rho \left(\vec{V}\cdot \nabla \right)\vec{V}=-\nabla \;\vec{P}+\eta \nabla \cdot \left[\nabla \vec{V}+{\left(\nabla \vec{V}\right)}^{T}\right]+\rho \vec{g}$$

where *t* is the time; ρ is the density; $\vec{V}$ is the velocity vector; $\vec{P}$ is the pressure vector; $\vec{g}$ is the gravity. However, the melting viscosity (η) is a function of temperature and shear rate for a non-Newtonian fluid of a melt polymer, which can also be found from Figure 2b. The function of viscosity can be taken as the Cross-WLF model, which is a popular and earlier alternative to the Bird–Carreau–Yasuda model [16]. The viscosity $\eta \;\left(\mathrm{kg}/\mathrm{mm}\;\sec.\right)$ is as

(2) η=η01+η0γ˙τ*1−n 

where $\tau^{*}$ is critical shear stress at the transition from the Newtonian plateau in viscosity with respect to the shear rate of non-Newtonian fluids; *n* is the power law index; *P* (dyne/mm^2^) is the pressure of the melt polymer; $\dot{\gamma }$ is the shear rate of fluid/; $\eta_{0}$ (kg/mm sec) is the viscosity under zero shear rate, η0=D1expA1Tc−TA2−Tc+T, in which $T_{c}=D_{2}+D_{3}P$ and $A_{2}={\tilde{A}}_{2}+D_{3}P$. The constants of viscosity of recycled PP used in this study have *n* = 0.225431, $\tau^{*}\;$= 8341.73 (dyne/mm^2^), *D*_1_ = 4.77 × 10^15^ (kg/mm sec), *D*_2_ = 263.15 (°K), *D*_3_ = 0 (°K mm^2^/dyne), *A*_1_ = 43.5548, and ${\tilde{A}}_{2}\;$ = 51.6 (°K).

In fluid dynamics, the continuity equation states that the rate at which mass enters a system is equal to the rate at which mass leaves the system plus the accumulation of mass within the system. The mass conservation of the melt polymer during the filling stage is described by a continuity equation. The differential form of the continuity equation [17] is as

(3) $$\frac{\partial \rho }{\partial t}+\nabla \cdot (\rho \vec{V)}=0$$

To describe the conservation of energy during the filling stages in the injection molding process, the energy equation in the vectors expression is as follows.

(4) $$\rho C_{p}\left(\frac{\partial \vec{T}}{\partial t}+\vec{V}\cdot \nabla \vec{T}\right)=\nabla^{2}(k\vec{T})+\eta (\nabla \times \vec{V})\cdot (\nabla \times \vec{V})+\rho \frac{d\alpha }{dt}∆H\;$$

where $C_{p}$ is the constant pressure specific heat time; $\vec{T}$ is the temperature distribution of the polymer within cavity of mold; *k* is the thermal conductivity; the shear rate in vector expression is $\vec{\dot{\gamma }}=\nabla \times \vec{V}$*;* and the shear stress induced by the viscosity and gradient of tangential velocity (shear rate) is $\vec{\tau }=\eta \vec{\dot{\gamma }}$. For semi-crystalline polymer flows, the release of latent heat due to crystallization is just like the heat source term in the energy equation, ∆H is the latent heat of fusion per unit mass for a perfect crystals of polymer, and α is the degree of crystallinity.

The temperature field ($\vec{T}$) of the plastic pallet after it is ejected from the mold is governed by the three-dimensional transient heat conduction equation, with constant properties. The equation is

(5) $$\nabla^{2}\vec{T}=\frac{\rho C_{p}}{K_{p}}\frac{\partial \vec{T}}{\partial t}$$

where $K_{p}$ is the coefficient of thermal conduction of plastic; ρ is density of polymer; $C_{p}$ is the constant pressure specific heat of polymer; and *t* is time in the mold plates. The boundary conditions of the demolded plastic under the natural convection in the atmosphere environment are

(6) $$K_{p}\nabla \;\vec{T}=-h(\vec{T}-{\vec{T}}_{a})$$

where ${\vec{T}}_{a}$ is air temperature around the pallet and h is the coefficient of natural thermal convection. ASTM standards (ASTM D955-08) have defined that the shrinkage is measured 24–48 h after demolding, when it has been subjected to the fully developed warpage of the molded part induced by inhomogeneous shrinkage. The shrinkage has been classified into three types [18]: (i) material anisotropy-induced shrinkage difference in different directions; (ii) non-uniform pressure and temperature distributions-induced shrinkage variations over the part; (iii) differential cooling effect on mold face-induced non-uniform shrinkage across the thickness. The cooling effects by natural convection acting on the ejected pallet in an ambient environment may cause non-uniform shrinkage across the thickness of pallet. The coefficient of heat convection under the pallet is smaller than the one above the pallet once the ejected pallet is on the ground, yielding additional warpage behind the ejection stage of injection molding.

## 4. Results and Discussion

### 4.1. Flow Front Comparison of Short-Shot Testing

The control system of the sequential valve gates activates the opening sequence of the gates, which can perform the relay filling of recycled PP. The activation sequence of the gates depends on the correct timing of the material flow. The flow front distribution timing can be estimated by a short-shot estimation during the actual filling. It can also be analyzed through the Moldex3D software by using the flow time of the filled recycled PP. Under simultaneous filling, the short-shot test is obtained by the ratio of the molding pallet weight obtained. The density of recycled PP of 1026 kg/m^3^ multiplies the volume of the plastic pallet of 13,160,000 mm^3^ with the addition of the chemical foaming agent. To determine the mass fill percent or the mass degree of the short-shot, the weight of the short-shot injection molded pallets is divided by the total pallet mass. The total pallet mass is found by taking the density multiplied by the total volume of the pallet, or by weighing the fully filled injection molded pallet.

**Figure 3:**
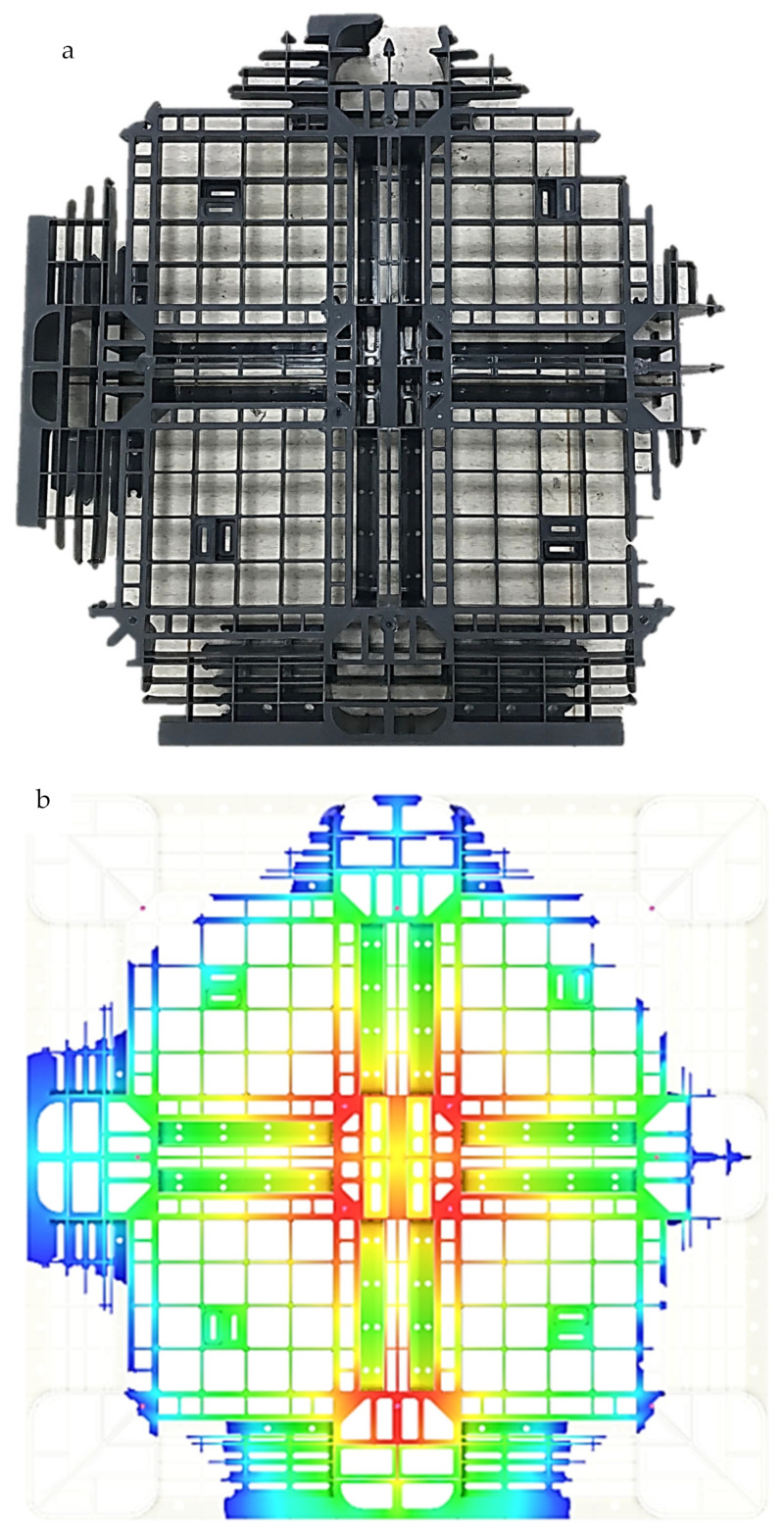

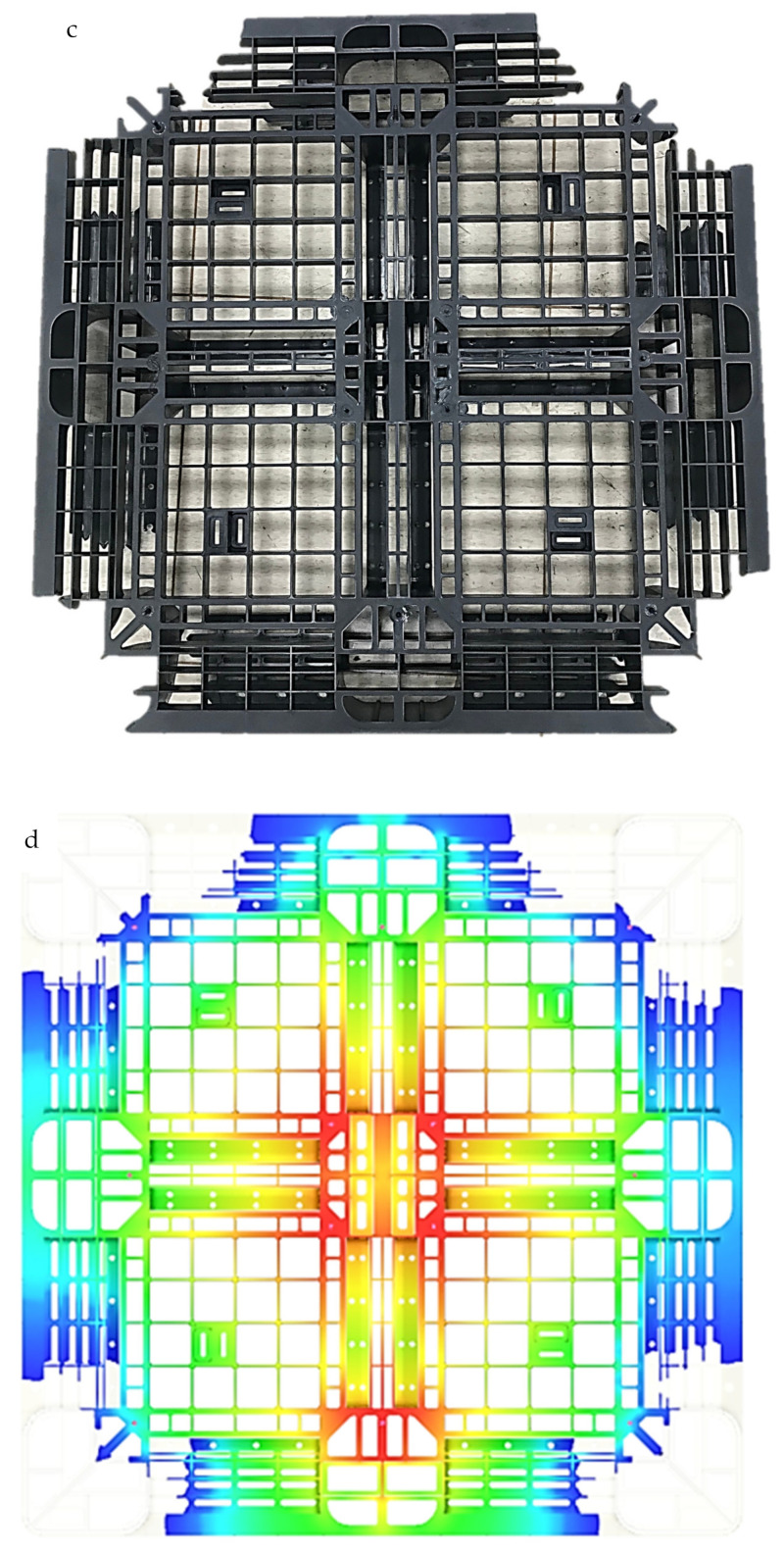
Comparisons of the experimental and numerical flow fronts in short-shot testing under the preset sequential valve gate scheme: (**a**) 57.9% short-shot true pallet; (**b**) 57.9% numerical flow front of short-shot testing; (**c**) 74.4% short-shot true pallet; (**d**) 74.4% numerical flow front of short-shot testing.

This mass fill percent can then be used to compare the experimental short-shot cases to the simulation, and the flow fronts can also be compared. In this study, the first short-shot molding pallet weight was 7.002 kg, which gives a short-shot ratio of 57.9%. The second short-shot molding pallet weight was 9.060 kg, giving a short-shot ratio of 74.4%. The flow front distribution is shown in Figure 3. Figure 3a shows that the time control is at 57.9% before the flow front, and as shown Figure 3b, the actual short-shot ratio is near 57.9%. Figure 3c shows that the time control is at 74.49% before the flow front, and as shown in Figure 3d, the actual short-shot ratio before the flow front is 74.49%. This shows the close relationships between the time control and actual flow front statistics.

### 4.2. Effects from Injection Molding Parameters

According to the time control for filling recycled PP, filling the melted material from different gates at various times would lead to different flow front styles. This can affect the contact and welding of the different flow fronts, and the melted materials’ weld lines, temperature, and location could affect the strength of the molded product. The flatness means the difference between the highest and lowest levels of the pallet surfaces. When goods are stacked on a pallet, to maintain stability, flatness should be decreased. To minimize warpage of a pallet, an optimization analysis is needed; the conditions include mold temperature, melt temperature, cooling waterway temperature, and cooling water temperature. By increasing the packing time and reducing the cooling time, the cycle time is unchanged during the analysis. In an injection molding experiment, cooling water temperature is considered to equal mold temperature. However, in our study the mold temperature is higher than the cooling water temperature, due to the fact that the heat within the mold has never been transferred away during the previous cycle of injection molding. A higher mold temperature reduces the difference between the temperature of the molded part and mold temperature, thus leading to a greater density.

**Figure 4:**
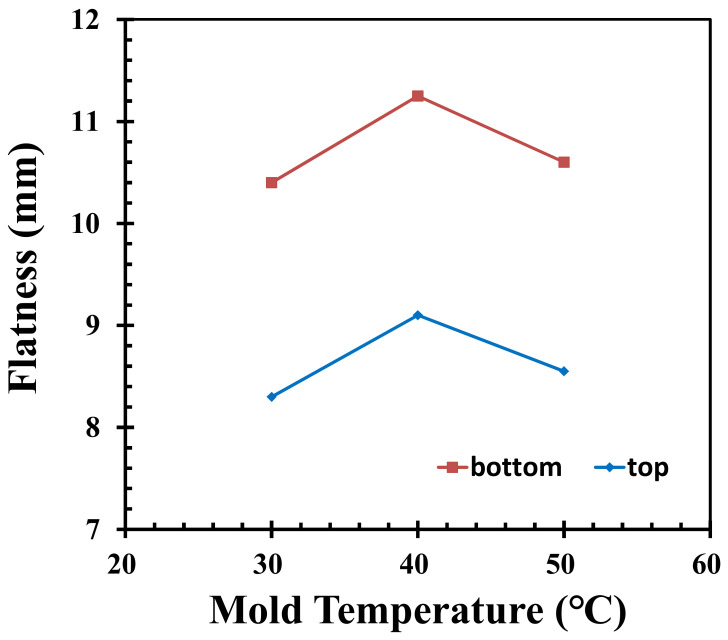
Flatness of the pallet when adjusting the mold temperature.

Under the fixed conditions of the other injection molding parameters, accordingly, the mold temperatures are 30 °C, 40 °C, and 50 °C. In Figure 4, the resulting flatness profiles of the top surface of the pallet are 8.3 mm, 8.5 mm, and 9.2 mm, respectively, while the flatness profiles of the pallet bottom surface are 10.4 mm, 10.6 mm, and 10.7 mm, respectively. On the one hand, as the mold temperature increases, the flatness of the top and bottom of the pallet also increases. This could be caused by the increase in mold temperature, which results in a longer freeze time and increases the effectiveness of the packing. On the other hand, the flatness of the top and bottom of the pallet decreases with respect to the mold temperature. This could be caused by the decrease in mold temperature, leading to a small difference in the shrinkage of the material. These two trends all produce benefits for the product; hence, the product may not show a positive or negative result. There is an overall 3.5% and 2.8% change in the flatness of the top and bottom of the pallet, respectively, when the mold temperature increases or decreases by 10 degrees.

**Figure 5:**
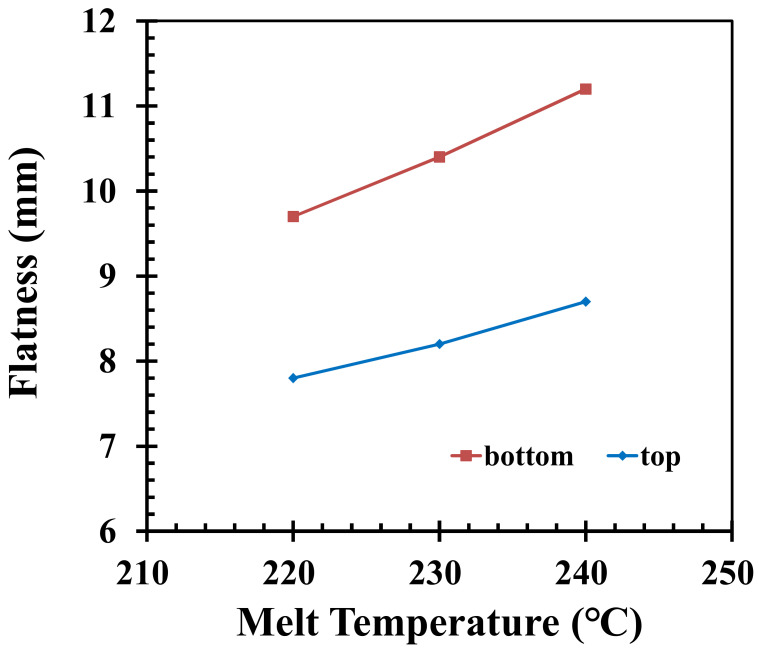
Influence on flatness values by adjusting the melt temperature.

A higher melt temperature under the same cycle time of the injection molding process may yield a higher average temperature of the pallet when the pallet is demolded. This is because the warpage only occurred after demolding from the mold; at that time, the pallet is free to stretch. Similarly, as shown in Figure 5, by fixing other injection parameters, where the melt temperatures are 220 °C, 230 °C, and 240 °C, the top surface areas of the pallet are 7.8 mm, 8.5 mm, and 9.2 mm, respectively, while the bottom surface areas of the pallet are 9.7 mm, 10.6 mm, and 11.4 mm, respectively. According to Figure 5, it can be seen that when the melt temperature is increased from 220 degrees to 240 degrees, the surface areas of the top and bottom of the pallet are increased. An increase in melt temperature leads to a larger difference in surface temperature; an increase in material mobility due to greater shrinkage leads to an increase in displacement; after the material cools down, the shrinkage decreases. When the displacement is small, an increase or decrease in temperature of 10 degrees affects the flatness of the top and bottom of the pallet by 15.2% and 14.9%, respectively.

**Figure 6:**
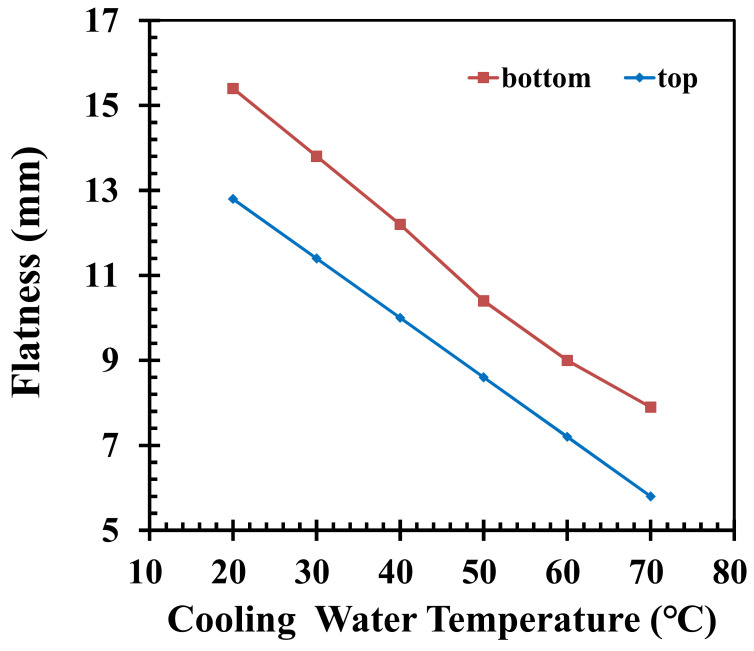
Influence on flatness values by adjusting the cooling water temperatures.

**Figure 7:**
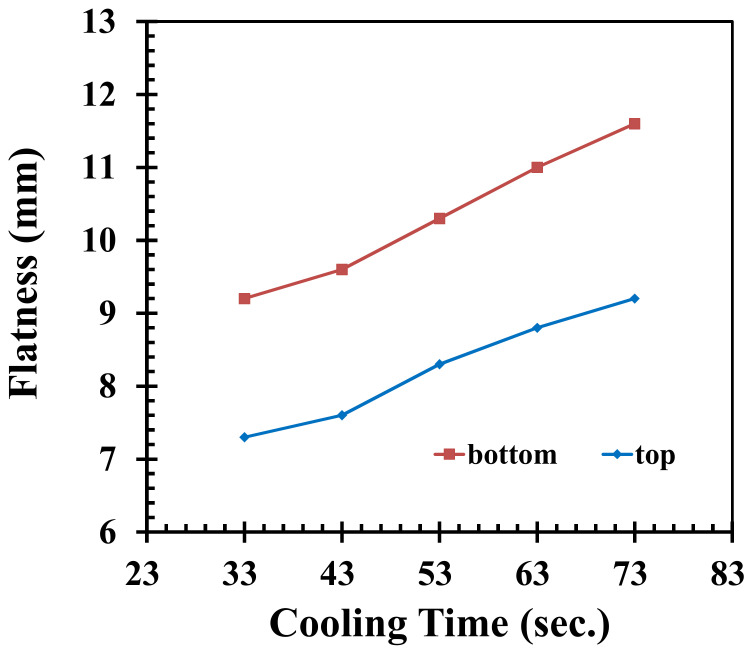
Influence on flatness by adjusting the cooling time.

The cooling water temperatures are set as 20, 30, 40, 50, 60, and 70 °C to analyze how the flatness of the product is affected. It can be seen in Figure 6 that when the temperature of the cooling water increases, the flatness values have a clear downward trend, while decreasing the temperature of the cooling water leads to higher flatness values. By increasing the temperature of the cooling water, the freeze time is lengthened, which leads to more effective packing. Due to the lower and upper limits of the cooling water temperature, which are 20 °C and 70 °C, respectively, the change in flatness values of the top and bottom of the pallet are 55.5% and 52.3%, respectively. The cooling time is 33, 43, 53, 63, and 73 s, while the other injection parameters are fixed according to Table 1. Through simulation, the flatness of the pallet is shown in Figure 7. As can be seen, when shortening the cooling time, the average temperature of the pallet is decreased while the pallet is ejected from the mold; therefore, the temperature difference between the highest and lowest point on the same surface is increased. This causes the flatness values of the top and bottom of the pallet to be increased. An increase in cooling time results in a temperature difference between the exterior and interior of the pallet, which leads to a higher rate of deformation in the z-axis. The warping of the product also increases. However, with a shorter cooling time, lower flatness values would result.

**Figure 8:**
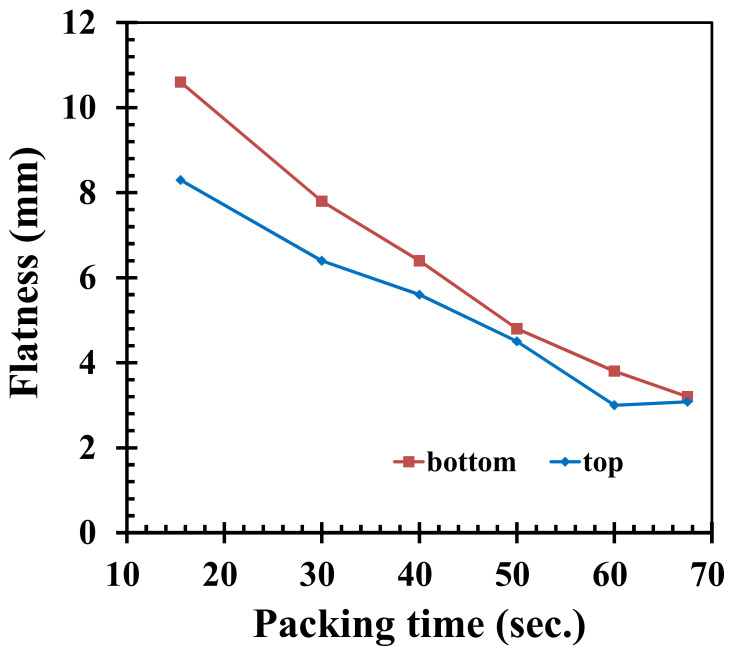
Influence on flatness when lengthening the packing time and shortening the cooling time.

A longer packing time may decrease the average temperature of the pallet, and thus decrease the associated warpage of the pallet. Under the condition that the total cycle time stays the same, while the packing time is increased and cooling time is decreased, when the time used is 68.5 s as in Figure 8, flatness would be affected by changes in these parameters. Therefore, the packing times are set at 15.5, 30, 40, 50, 60, and 67.5 s, according to Table 3. Figure 8 shows how the flatness values decrease with an increase in packing time. The clamping force and the final flow front of the material near the gates is affected more by a lengthened packing time, resulting in an increase in flatness. Under the same cycle time, while taking the upper limit of the packing time, changes in the flatness values of the top and bottom of the pallet are 65.9% and 67.9%, respectively. If the packing time of 40 s is increased or decreased by 10 s, the change in flatness values of the top and bottom of the pallet are 33.3% and 39.7%, respectively.

### 4.3. Flatness of Pallet Using the Preset Sequential Scheme

Following Table 2, the actuating times of all of the gates are listed in Table 3, where start and stop times to fill each gate are easily described and compared. The gates of 5, 9, and 10 are abnormally switched using the machine manufacturer’s preset sequence. By using the time control of the 9 phases, phase 1 refers to the opening of gates 1–4 and 10 at 0 s. Phase 2 is the closure of gates 1–4 after 6 s. During phase 3, gates 5 and 9 continue the filling process. Phase 4 is the opening of gate 8 after 9 s, and phase 5 closes gates 5, 9, and 10. Phase 6 occurs at 9.7 s, where gate 9 is opened with an interval of 0.1 s, and phases 8 and 9 refer to the opening of gates 5 and 10, respectively. Finally, phase 9 is the opening of gates 6, 7, 11, and 12 to finish the filling process until completion.

**Figure 9:**
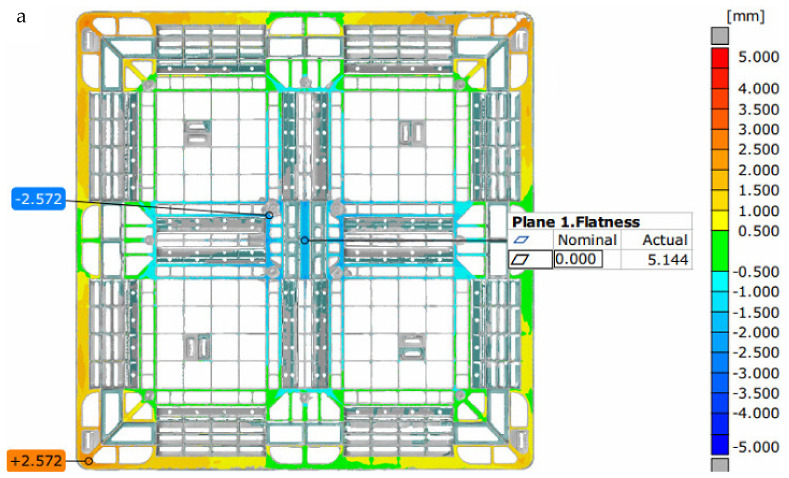

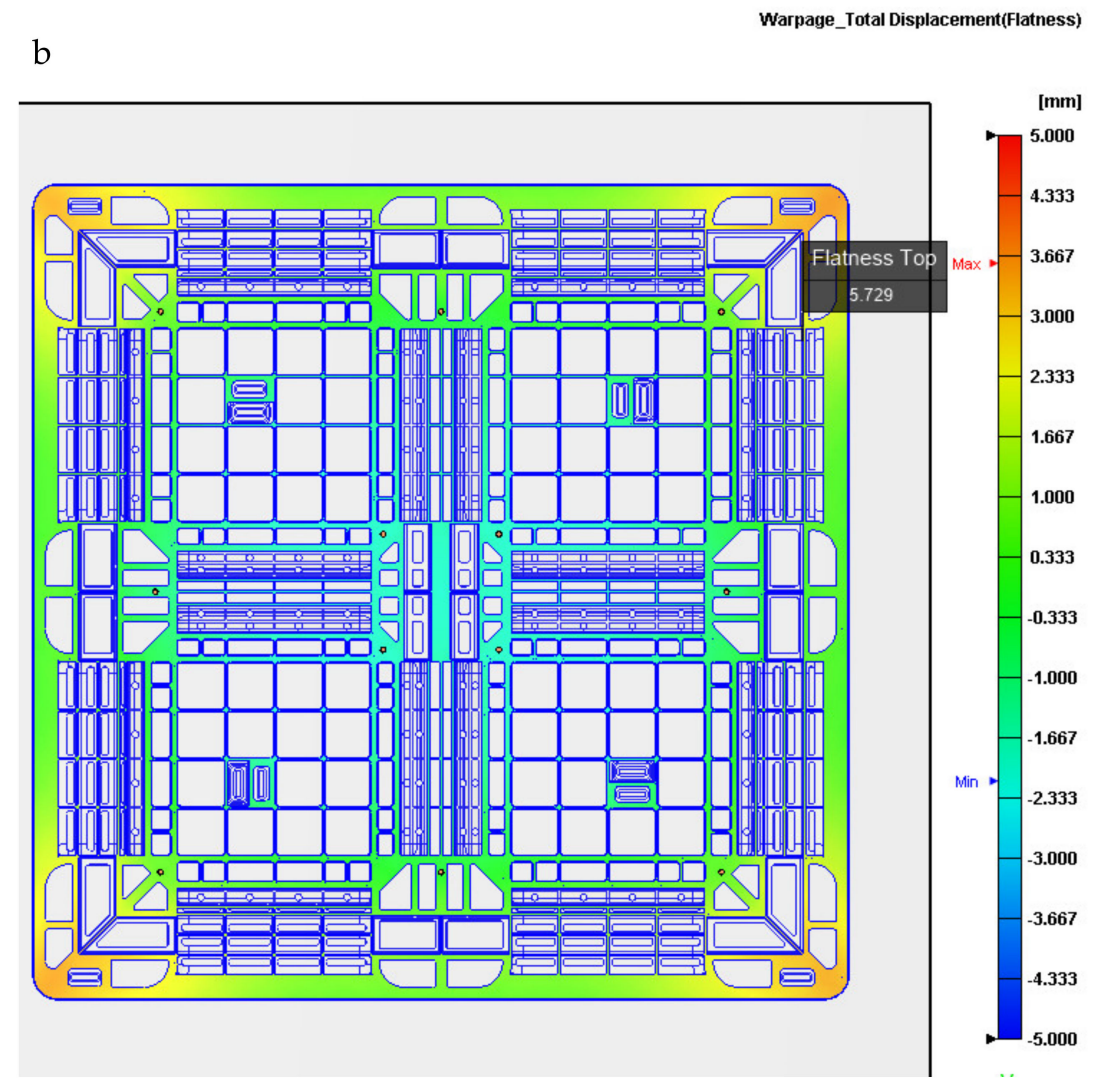
(**a**) Changes in the top height of the pallet measured by the ATOS system (flatness 5.144 mm); (**b**) changes in the top height of the pallet under the time control of 40 s of packing time (flatness 5.729 mm).

Under the preset sequence of the filling valve gates, as depicted in Table 3, the contribution volume of each gate depicts that gates 6, 7, 11, and 12 are the major contributors in filling the melt polymer into the mold cavity; their contribution volumes reach about 50% of the total volume of the pallet. Gates 5, 9, and 10 have interruptions on filling, which may induce mechanical faults on the gates. At the initial filling stage by gates 1 to 4, the filling times are 6 s, which provide larger flow lengths from the gates for this rib-structured pallet. The top surface profile of the actual pallet measured by the ATOS scan system is displayed in Figure 9a, which is concave and has a flatness of 5.144 mm. The numerical top surface profile of the pallet derived by the Moldex3D 2020 is shown in Figure 9b, where the flatness is 5.729 mm and matches the actual flatness.

### 4.4. Flatness Verifications by Proposed Sequential Scheme

**Figure 10:**
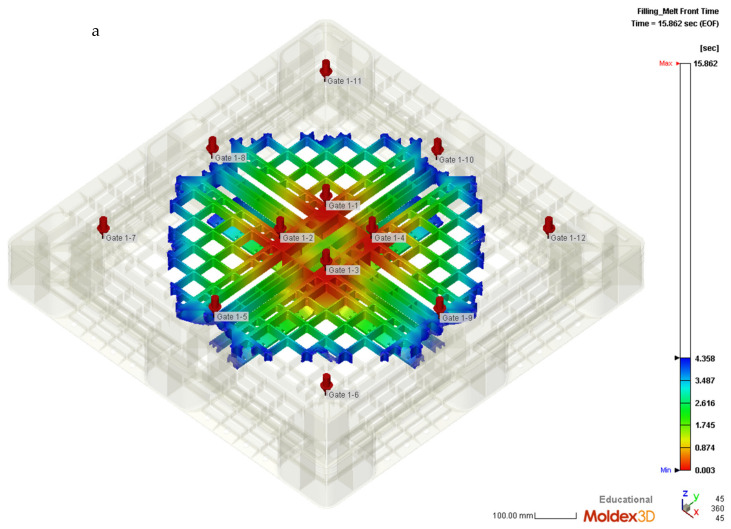

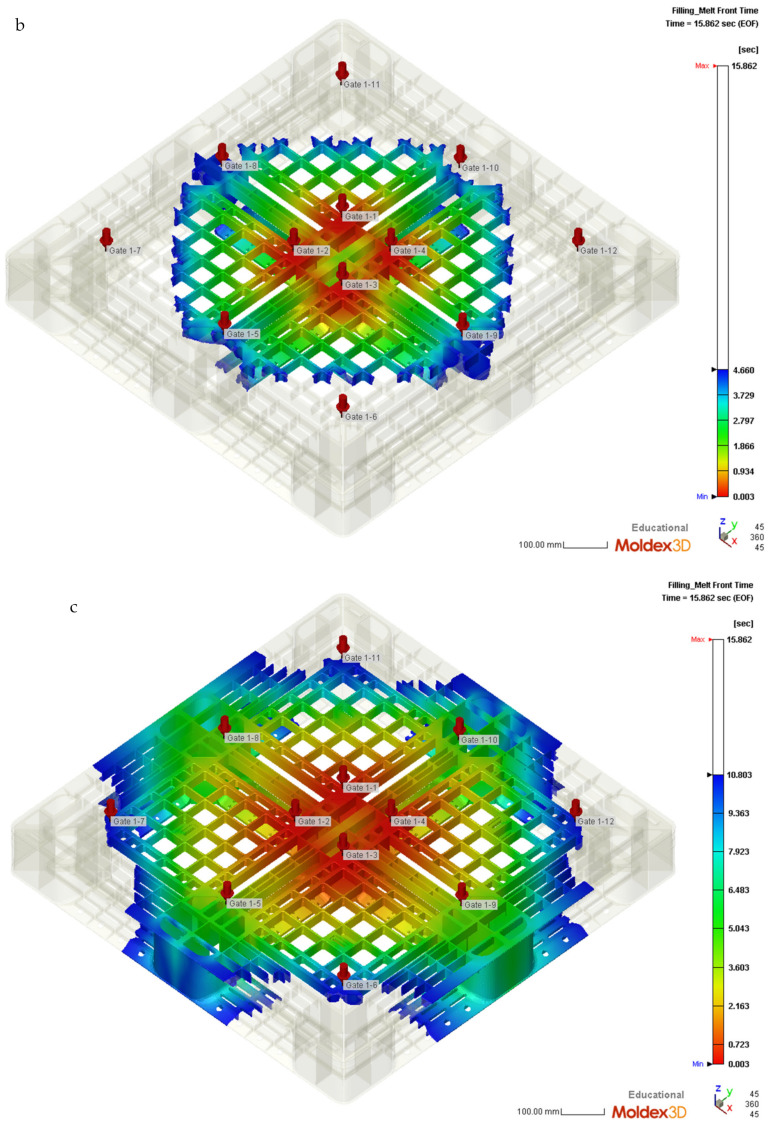
(**a**) Flow front spreading to hit gates 5 and 9 at 4.35 s; (**b**) Flow front spreading to hit gate 8 at 4.67 s; (**c**) Flow front spreading to hit gates 6, 7, 11, and 129 at 10.8 s.

By using the propagations of flow fronts from the filling gates via simulations on Moldex3D 2020, Table 4 shows the new proposed sequence of the valve gates replacing those shown in Table 2. The simulated flow fronts are from the initial opening of gates 1 to 4 during the filling stage. Gates 5 and 9 are then opened once, which meets the initial flow front at 4.35 s, as shown in Figure 10a. Then, in Figure 10b, gates 8 and 10 are opened at the times of 4.67 and 4.72 s, respectively. Figure 10c depicts that at the time of 10.8 s, all the valve gates are actuated to fill the melt polymer. All the valves of gates are closed until the end of the packing stage. Under the scheme of setting the valve gate actuating to open once the flow front spreads to it, the actual warpage of the pallet was measured to compare with the numerical values. Table 5 indicated the switiching time of each filling gate. The valve gates would be divided into three categories. The gates of number 5, 9, 8 and 10 are included to the second category for different reaching time of the initial flow front to them. All the valve gates are closed before packing stage of injection molding process.

**Figure 11:**
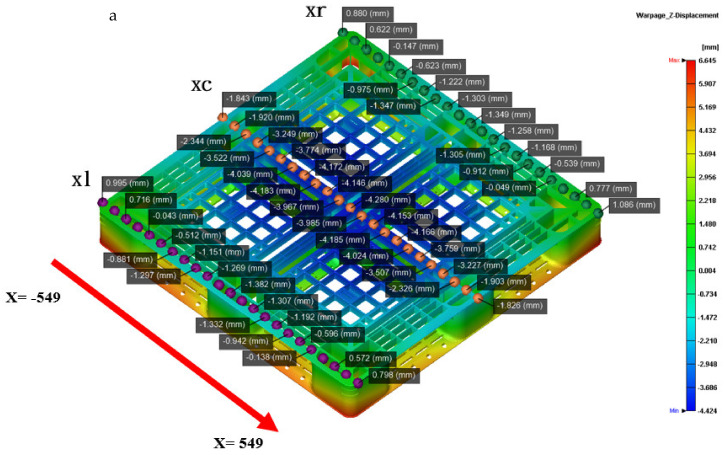

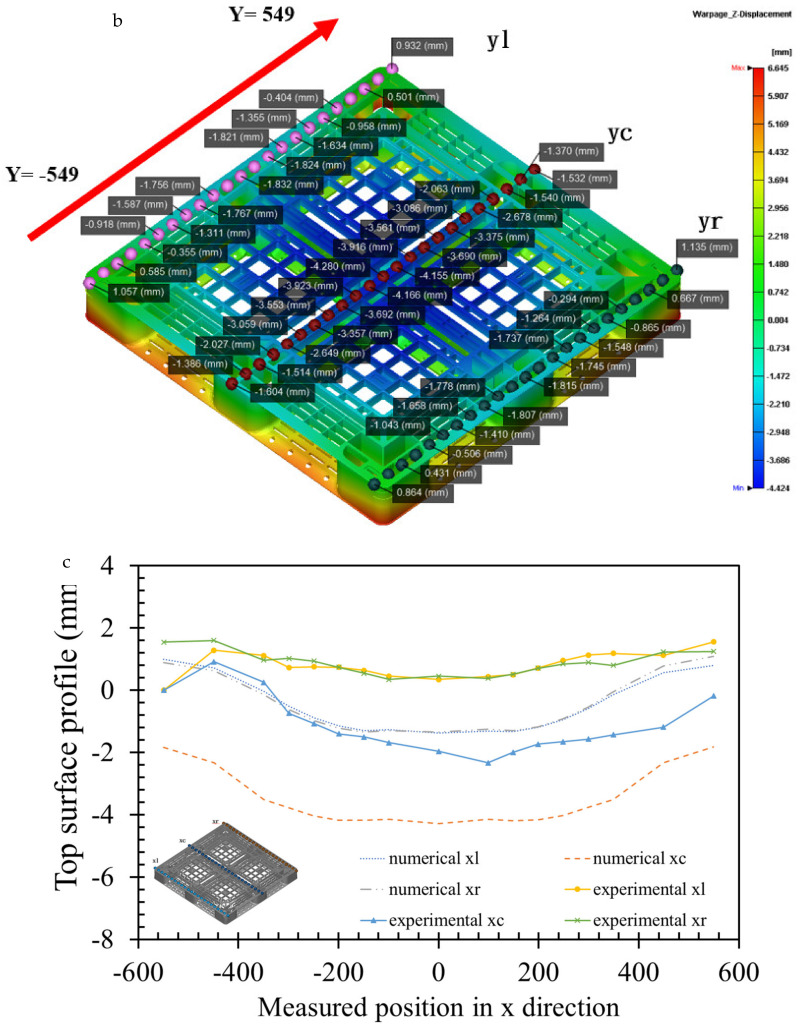

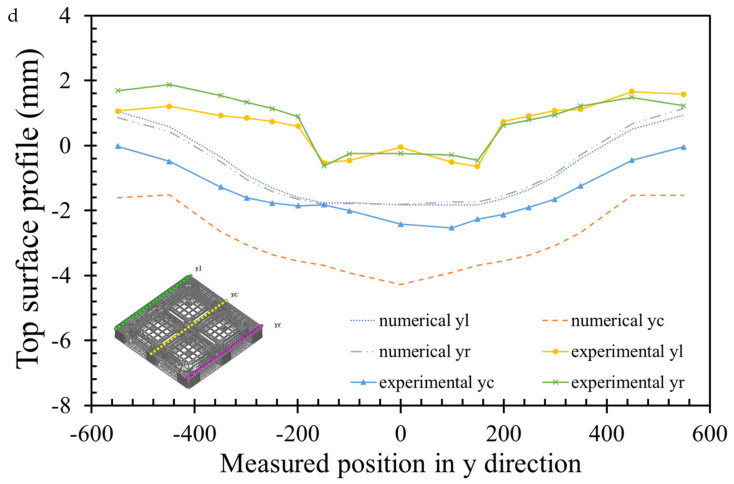
(**a**) Measured point for the top height of the pallet in the x-axis direction; (**b**) measured point for the top height of the pallet in the y-axis direction; (**c**) comparison between the simulated and actual top height profiles of the pallet in the x-axis direction; (**d**) comparison between the simulated and actual top height profiles of the pallet in the y-axis direction.

Figure 11a shows that the actual flatness of the top of the pallet is 5.1 mm, and Figure 11b shows that the simulated flatness of the top of pallet is 5.8 mm, under the usage that the packing time by time control is 40 s. The schematic diagrams showing the measurement positions of the height deformation along the x- and y-axis of the actual and simulated top pallet surface are shown in Figure 11a,b, while the warping trends of the height deformation along the x- and y-axis are shown in Figure 11c,d. Through the simulated and actual height deformations of the top pallet surface, it can be seen that the outer part warps upwards, while the central part warps downwards.

From the above analysis, we can draw conclusions about how the injection parameters can affect flatness. By adjusting the temperature parameters, the greatest impacts on flatness occur by changing the cooling water temperature, material temperature, and mold temperature, whereas by adjusting the time parameters, the greatest impacts on flatness occur by increasing the packing time and cooling time. Therefore, the injection parameters that have an impact on flatness are packing time, cooling water temperature, material temperature, cooling time, sequential valve gate control, and mold temperature. On the basis of the analysis, increasing the packing time to 40 s and reducing the cooling time, while maintaining the cycle time, results in the simulated flatness being closer to the actual flatness. The simulated flatness of the top and bottom pallet surface is 5.7 mm and 6.4 mm, respectively, while the actual flatness of the top and bottom pallet surface is 5.1 mm and 6.4 mm. Since the results of the simulated pallet are remarkably close to that of the actual pallet, the simulated results can be used as a reference.

**Figure 12:**
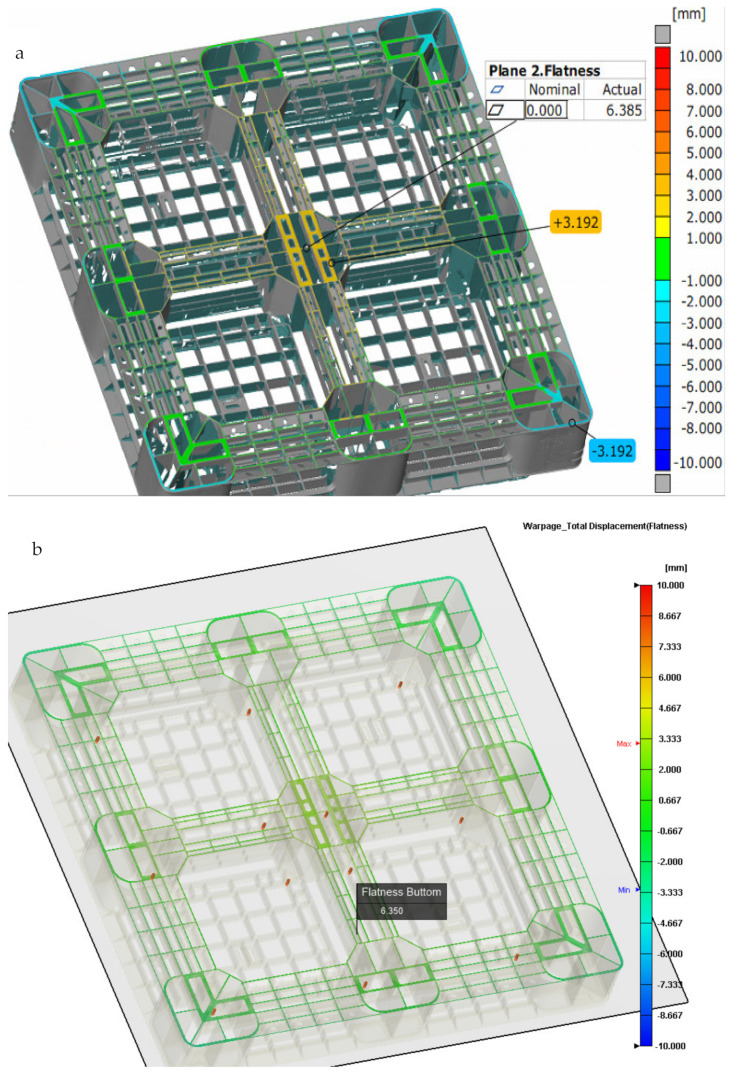
(**a**) The actual height profile change of the bottom side of the pallet measured by the ATOS system (flatness 6.385 mm); (**b**) simulated changes of the bottom height profile of the pallet during a packing time of 40 s by time control.

**Figure 13:**
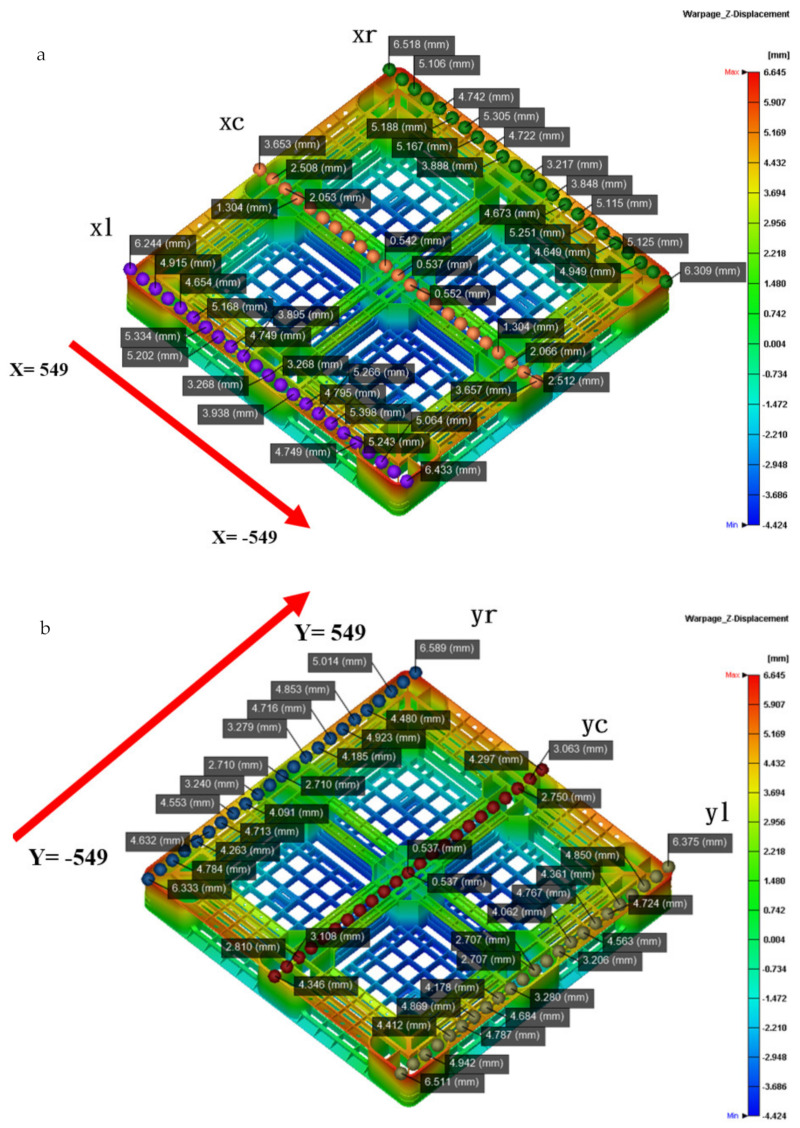

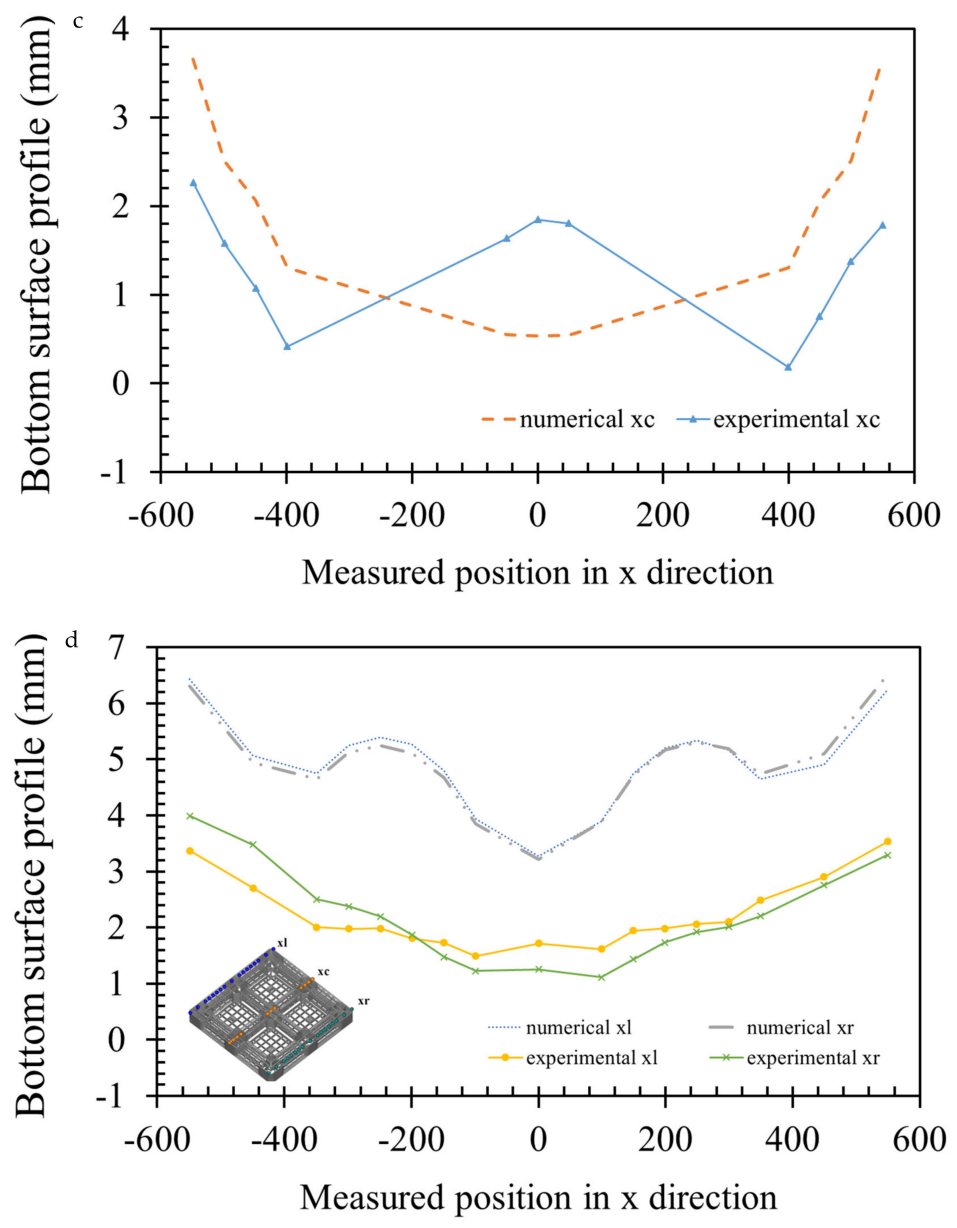
(**a**) Bottom side height profile measurement points in the x-axis direction of the pallet; (**b**) bottom side height profile measurement points in the y-axis direction of the pallet; (**c**) comparison of the simulated and actual heights of the bottom central part of the pallet in the x-axis direction; (**d**) comparison of the simulated and actual bottom side height profiles of both sides of the pallet in the x-axis direction.

As shown in Figure 12a, the actual bottom flatness of the pallet by the ATOS scan system is 6.385 mm under the proposed sequential valve gate scheme. Figure 10b shows that the simulated bottom flatness of the pallet, when using a packing time of 40 s by time control, is 6.350 mm. The schematic diagrams showing the measurement positions of the height deformation along the x- and y-axis of the actual and simulated top pallet surface are shown in Figure 13a,b. Figure 13c,d displays the height deformation in the x-axis. While an upward warpage trend can be seen in the simulated central part along the y-axis, in the actual product, a downward trend is displayed. The deformations in the actual and simulated products differ by around 1.3 mm. The simulated and actual measurement positions on both sides of the x-axis show the same trend, while the center concaves upward. This analysis meets the actual flatness, and the top flatness of the pallet is closer to the actual warpage trend, as compared with the bottom flatness of the pallet. To improve the accuracy of the numerical prediction, we need to perform more experiments in the future.

According to the results of the analysis above, the injection parameters can affect the flatness of the pallet. By adjusting the temperature parameters, which are the cooling water temperature and the mold temperature, the influence is greater than that obtained by adjusting the time parameters, which are increasing the packing time and cooling time. The injection parameters that have the largest impact on flatness are the packing time, cooling water temperature, material temperature, cooling time, sequential scheme, and mold temperature. Through analysis, the actual and simulated flatness of the pallet are closest by increasing the packing time to 40 s and reducing the cooling time, while keeping the length of the cycle. Under these conditions the simulated top and bottom flatness of the pallet are 5.7 mm and 6.4 mm, respectively, while the actual top and bottom flatness of the pallet are 5.1 mm and 6.4 mm, respectively. As the results of the simulated pallet are close to that of the actual pallet, the simulated results can be used as a reference. The deformation profile in the height direction shows a deformation trend that concaves downwards. This warping trend is seen from both the cross section and the exterior.

## 5. Conclusions

This research uses mold flow analysis software to simulate the results of injection molding. By using time control to simulate the flow front of the pallet, the simulated material flow can be similar to that of an actual flow situation. The influence of the injection molding parameters on the molding is also discussed. Through simulation analysis, the simulation flow that is closest to the actual flow situation of the pallet is found to be provided by using sequential time control. By using nine phases of time control, the short-shot ratio of the opening and closing of the sequential valve gates are 57.9% and 74.5%. An increase in packing time and decrease in cooling time, while keeping the same cycle time, adjusting the cooling water temperature, increasing the mold temperature, and decreasing the material temperature, can significantly reduce the overall flatness and warpage displacement. When the packing time is 40 s, the flatness of the simulated pallet is closest to that of the actual pallet. The difference in top flatness between the simulated and actual pallet is 0.59 mm, while the bottom flatness difference is 0.035 mm. The greatest influences on the flatness of the pallet are the packing time, cooling water temperature, material temperature, cooling time, sequential valve gates control, and mold temperature.

## Figures and Tables

**Table 1:** Injection molding parameters of recycled polypropylene [14].

Parameter	Setting
Melt temperature (°C)	250
Freeze temperature (°C)	117
Mold temperature (°C)	45
Injection rate (g/s)	1782
Injection pressure (MPa)	40.8
Filling time (s)	15.0
Packing time (s)	15.5
Packing pressure (MPa)	30.7
Cooling time (s)	53
Mold opening time (s)	20

**Table 2:** Preset sequence of the filling valve gates.

Phase	1	2	3	4	5	6	7	8	9
Time (s)	Start	6.0	8.0	9.0	9.7	9.8	9.9	12.0	15.0
Gate opening	1–4, 10	5, 9	8		9	5	10	6, 7, 11, 12	
Gate closing		1–4		5, 9, 10					5–12

**Table 3:** Switching times of the preset sequence of the filling valve gates.

Sequence of the Valve Gates													
	Time	0.0	1.0	3.0	6.0	8.0	9.0	9.7	9.8	9.9	12	15	Contribution Volume
Gate													
1		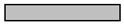											4.60%
2		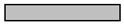											4.61%
3		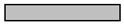											4.60%
4		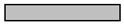											4.61%
5					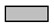				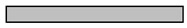				6.54%
6											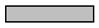		12.37%
7											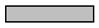		12.43%
8						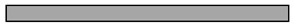							9.43%
9					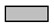			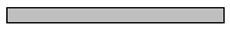					9.44%
10		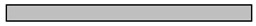								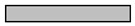			6.56%
11										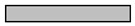			12.40%
12										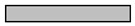			12.42%

**Table 4:** Proposed sequence of the filling valve gates.

Phase	1	2	3	4	5
Time (s)	Start	4.35	4.67	4.72	10.8
Gate opening	1–4	5, 9	8	10	6, 7, 11, 12

**Table 5:** Switching times for the proposed sequence of the filling valve gates.

Sequence of the Valve Gates													
	Time	0.0	1	3	4.35	4.67	4.72	6	8	10.8	12	15	Contribution Volume
Gate													
1		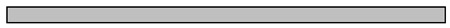											6.49%
2		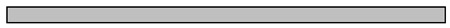											6.97%
3		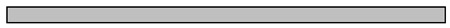											6.97%
4		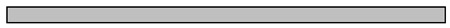											6.49%
5					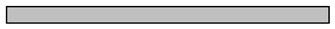								9.10%
6										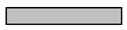			6.65%
7										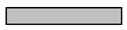			7.47%
8						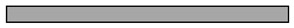							12.64%
9					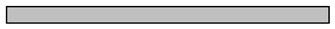								14.59%
10							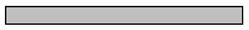						12.63%
11										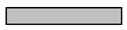			5.27%
12										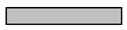			4.74%

## Data Availability

All the data generated or analyzed during this study are included in the published article.
